# Tussilagone Suppresses Angiogenesis by Inhibiting the VEGFR2 Signaling Pathway

**DOI:** 10.3389/fphar.2019.00764

**Published:** 2019-07-05

**Authors:** Jia Li, Jiangtong Peng, Shengnan Zhao, Yi Zhong, Yilong Wang, Ji Hu, Chao Zhang, Min Cheng, Geqing Xia, Yu Hu, Kai Huang, Yan Wang, Minglu Liang

**Affiliations:** ^1^Clinic Center of Human Genomic Research, Union Hospital, Tongji Medical College, Huazhong University of Science and Technology, Wuhan, China; ^2^Department of Cardiology, Union Hospital, Tongji Medical College, Huazhong University of Science and Technology, Wuhan, China; ^3^Department of Vascular Surgery, Union Hospital, Tongji Medical College, Huazhong University of Science and Technology, Wuhan, China; ^4^Institute of Pathology, Tongji Hospital, Tongji Medical College, Huazhong University of Science and Technology, Wuhan, China; ^5^Department of Obstetrics and Gynecology, Union Hospital, Tongji Medical College, Huazhong University of Science and Technology, Wuhan, China; ^6^Institute of Hematology, Union Hospital, Tongji Medical College, Huazhong University of Science and Technology, Wuhan, China

**Keywords:** angiogenesis, TUSSILAGONE, VEGFR2 signaling pathway, human umbilical vascular endothelial cell, vascular endothelial growth factor

## Abstract

Tussilagone (TSL) is a sesquiterpenoid isolated from *Tussilago farfara*, which has been used as a traditional medicine for the treatment of asthma and bronchitis. It also takes part in the anti-inflammatory and antioxidant effects, but its role in angiogenesis is unknown. Angiogenesis is a cancer feature that is essential for supplying oxygen and nutrients to all proliferating tumor cells. Here, we demonstrated that TSL significantly inhibited the proliferation, migration, invasion, and tube formation of primary human umbilical vascular endothelial cell (HUVEC) *in vitro*. Also, TSL inhibited vascular endothelial growth factor (VEGF)-induced angiogenesis revealed by Matrigel plug assay *in vivo*. At present, we observed that TSL inhibited the activity of VEGFR2 signal pathway induced by VEGF. These findings suggested that TSL may serve as a potential therapeutic target in the angiogenesis.

## Introduction

Angiogenesis means new blood vessels formation, a process from preexisting vessels, including migration, proliferation, and formation of capillary tubes steps in endothelial cells. It is crucial for organ development and wound healing under physiological condition (Kerbel and Folkman, 2002; Jubb et al., 2006; Kunimasa et al., 2011; Lam et al., 2012). However, it also takes part in a series of diseases, such as diabetic retinopathy, peripheral vascular disease, endometriosis, tissue regeneration, atherosclerosis, obesity, rheumatoid arthritis, and cancer (Piao et al., 2018). Vascular endothelial growth factors (VEGFs), which are also characteristic of migration, proliferation, and tube formation in endothelial cells, are regarded as the main regulator of pathological angiogenesis with the receptors (VEGFRs), PIGF, angiopoietin-1/2, TSP-1/2 (Li et al., 2017).

**Figure 1:**
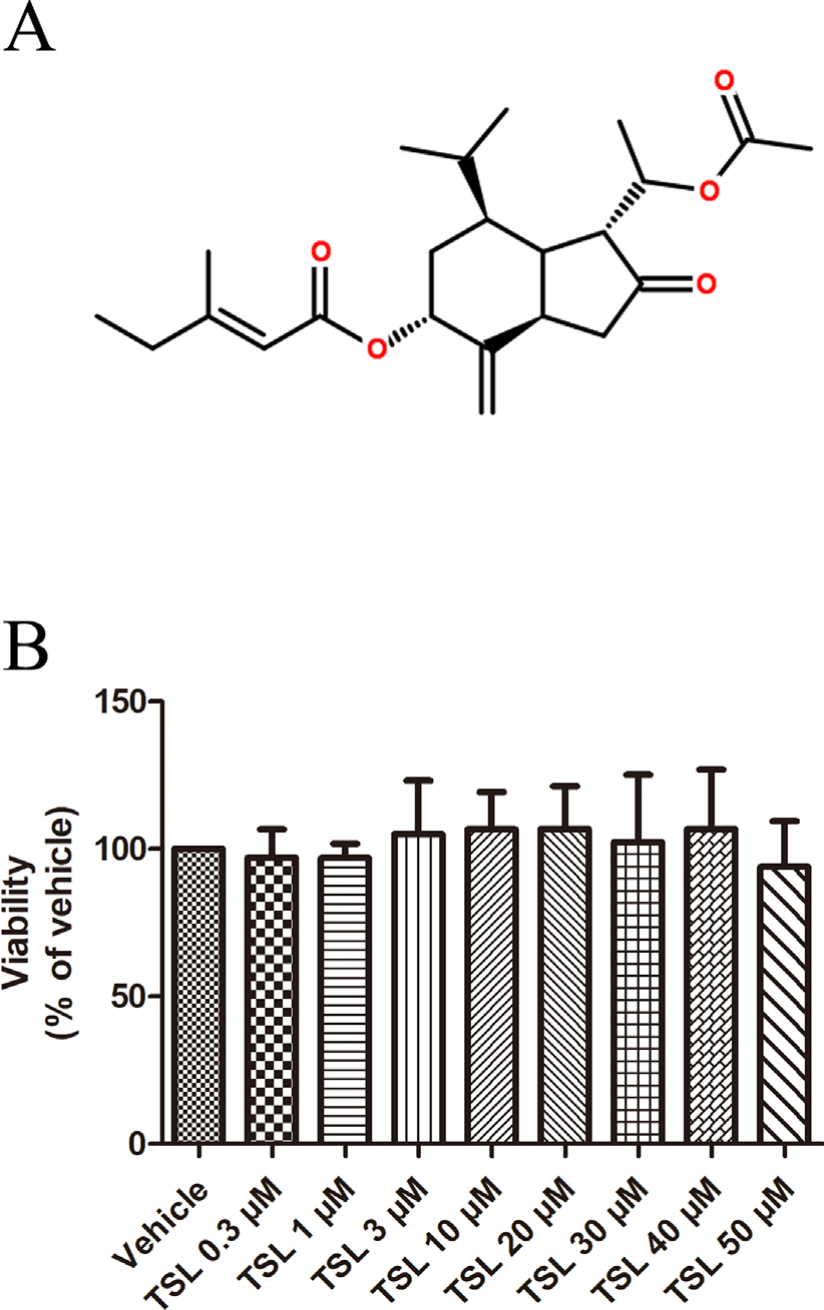
**(A)** Chemical structures of TSL. **(B)** The effect of TSL on cell viability. HUVECs were incubated with various concentrations of TSL for 24 h, and the cell viability was determined by MTT assay. Data are represented as mean ± SEM (n = 3).

Tussilagone (TSL) (**Figure 1A**) is the main ingredient extracted from flower buds of *Tussilago farfara* L. (also known as Kuandonghua or flos farfarae), which has been used for the treatment of asthma and bronchitis as a traditional medicine (Kim et al., 2017). According to previous studies, they have proven that TSL played a role in anti-inflammatory activity because of its antioxidant effect, which is associated with the decrease of nitric oxide (NO), tumor necrosis factor (TNF)-α, and prostaglandin E2 (PGE2) production in different cells, such as murine macrophages, dendritic cells, and microglial cells, stimulated by LPS (Lim et al., 2008; Hwangbo et al., 2009; Park et al., 2014). TSL participated in fat metabolism by inhibiting the synthesis of microsomal DGAT1 and triglyceride (Park et al., 2008). Furthermore, TSL showed antimicrobial activity and exhibited the suppression effects of platelet activating factor receptor and colon cancer cell proliferation (Hwang et al., 1987; Kokoska et al., 2002; Li et al., 2014).

As always, angiogenesis inhibition was perceived as a target therapy of angiogenesis-related diseases. Here, we investigated the effects of TSL on anti-angiogenesis in human umbilical vein endothelial cells (HUVECs) and its underlying mechanisms.

## Materials and Methods

### Reagents

TSL was purchased from Pufei De Biotech (Chengdu, China). Endothelial cell medium (ECM) was purchased from Sciencell (USA). Fetal bovine serum (FBS) was purchased from PAN Biotech (Germany). Recombinant human vascular endothelial growth factor (VEGF165) was obtained from PeproTech (USA). Matrigel was purchased from BD Biosciences (San Jose, CA, USA). EdU kit was purchased from Ribobio (GuangZhou, China). Anti-phospho-VEGFR2, anti-VEGFR2, anti-phospho-JNK, anti-JNK antibody, anti-phospho-ERK1/2, anti-ERK1/2 antibody anti-phospho-p38, anti-p38 antibody, anti-CDK2, anti-CDK4, and anti-tubulin were purchased from Cell Signaling Technology (USA). Anti-MMP2 and anti-MMP9 were purchased from Abcam (UK).

### Cell Culture

Primary HUVECs were cultured in ECM containing 10% FBS, 1% endothelial cell growth supplement, and 1% penicillin/streptomycin solution in 5% CO_2_ at 37°C. Passages 3 to 7 of HUVEC cells were used in all experiments.

### MTT Assay

The HUVECs were seeded into 96-well plates (5 × 10^3^ cells/well) and stimulated with various concentrations of TSL in 5% CO_2_ at 37°C with 2% FBS for 24 h. After incubation for 4 h with MTT dye (0.5 mg/ml), the culture medium was removed and replaced with 150 µl of dimethyl sulfoxide (DMSO). Followed by 10-min shaking at room temperature (RT), the absorbance was read at 570 nm by a microplate reader, and proliferation rate was calculated for each group. 

Based on the results in **Figure 1B**, 3-, 10-, and 30-μM TSL represented the optimal working concentrations used in our subsequent experiments. 

### EdU Incorporation Assay

The HUVECs were seeded into 96-well plates (1.2 × 10^4^ cells/well) and pretreated with indicated concentrations of TSL for 30 min in starvation, then stimulated with VEGF (25 ng/ml) for another 12 h. After incubation with a medium containing EdU for another 2 h, the cells were fixed with 4% paraformaldehyde and permeabilized with 0.1% Triton X-100 for immunostaining with Hoechst. Images were visualized and photographed using Olympus cellSens Entry. Each treatment was performed in triplicate.

### Would Healing Assay

The HUVECs were cultured into six-well plates up to 80% confluence. Cell monolayers were wounded with a sterile 10-μl pipette tip and washed with PBS for twice. Cells were then cultured in M199 plus 2% FBS and stimulated with TSL at various concentrations in the absence or presence of VEGF (25 ng/ml) for 48 h. Cells were photographed using Olympus cellSens Entry, the rate of cell migration was determined by the area of migration into the scratch using an Image J program. 

### Tube Formation Assay

The HUVECs were incubated into 96-well plates with indicated TSL at various concentrations for 6 h. Cells were then seeded on Matrigel in the presence of VEGF (25 ng/ml) with or without TSL. Tube formation was visualized and photographed using Olympus cellSens Entry. Tube-forming ability was quantified by counting the total number of branched tubes in selected visions with Image J. Each treatment was performed in triplicate, and five visions were counted to obtain an average.

### Western Blotting

The HUVECs were seeded into six-well plates up to 80% confluence. After being pretreated with indicated concentrations of TSL for 30 min, the cells were stimulated by VEGF (25 ng/ml) for another 5 min or 24 h. Western blotting was performed according to the procedures described previously (Zhao et al., 2019). All Western blot experiments were carried out at least three times.

### Matrigel Plug Assay

All animals were purchased from Tongji Medical College, Huazhong University of Science and Technology University. All experimental protocols were approved by the Ethics Committee of Tongji Medical College, Huazhong University of Science and Technology, and were performed in accordance with relevant institutional and national guidelines and regulations. Briefly, C57BL/6 mice were subcutaneously injected with 500-μl BD Matrigel Matrix and 50-unit/ml heparin mixture in presence or absence of VEGF (100 ng/ml). The mice were daily given a gavage with corn oil as control, TSL (1 and 10 mg/kg) and cabozantinib (XL184) 30 mg/kg. After 7 days, the Matrigel plugs were removed from the euthanized mice and fixed with 3.7% formalin in PBS and embedded in paraffin, then plugs were cut into sections. The sections were stained with hematoxylin and eosin (H&E) solutions for microscopic observation.

### Statistical Analysis

Statistical analyses were carried out using Graphpad Prism software (GraphPad Software, Inc., San Diego, CA). Student’s *t* test was used to test signiﬁcant differences between samples.

## Results

### TSL Suppressed the Proliferation of HUVECs Induced by VEGF

We first investigated whether TSL played a role in the process of angiogenesis in HUVECs. The MTT assay was designed to determine the appropriate dose of TSL, and the optimal concentrations were 3, 10, and 30 μM, which showed no obvious cytotoxicity to the cells (**Figure 1B**).

**Figure 2:**
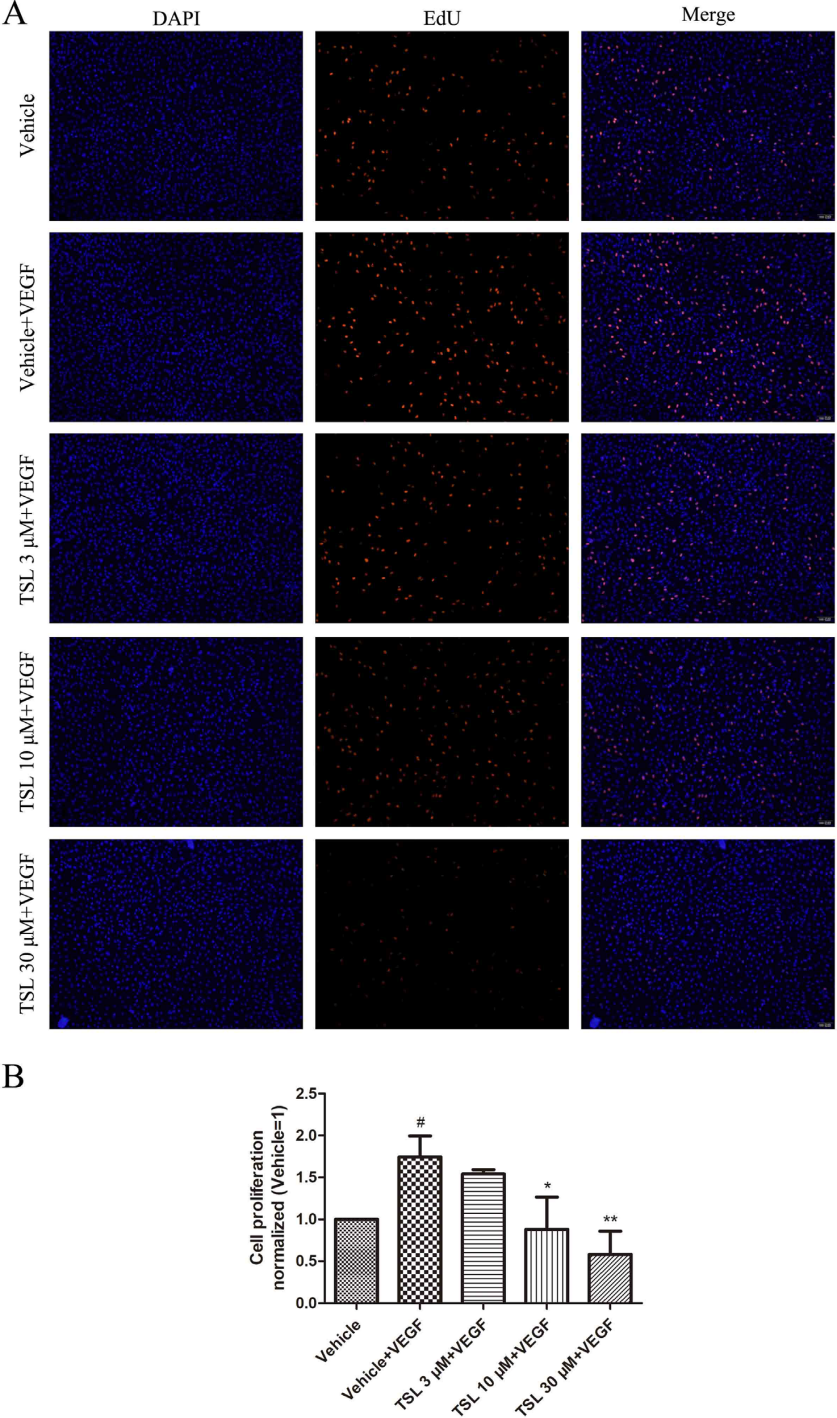
TSL inhibits VEGF induced HUVEC proliferation. **(A)** HUVECs were pretreated with indicated concentrations TSL for 30 min, then VEGF (25 ng/ml) was added into each group for 12 h. The cells were stained with EdU (red) and DAPI (blue). **(B)** Quantification of the proliferation cell (normalized to vehicle). Data are represented as mean ± SEM (n = 3). ^#^ *P* < 0.05 versus the vehicle group; *P < 0.05, **P < 0.01 versus the vehicle + VEGF group.

DNA synthesis was detected by EdU kit, the results indicated that the cell proliferation ability was apparently improved under the stimulation of VEGF, which was inhibited by TSL. The effect was presented in a dose-dependent manner of TSL, such as 3 μM of TSL, which showed no influence on cell proliferation, whereas both 10 and 30 μM of TSL suppressed the cell proliferation, especially at the 30-μM concentration (**Figure 2**). For further verification, cabozanitib (XL184), which is an effective inhibitor of VEGFR2, suppressed cell proliferation significantly at a concentration of 5 µM, but the effect was weaker than 30 μM in the TSL group (**Supplement Figure 1**).

**Figure 3:**
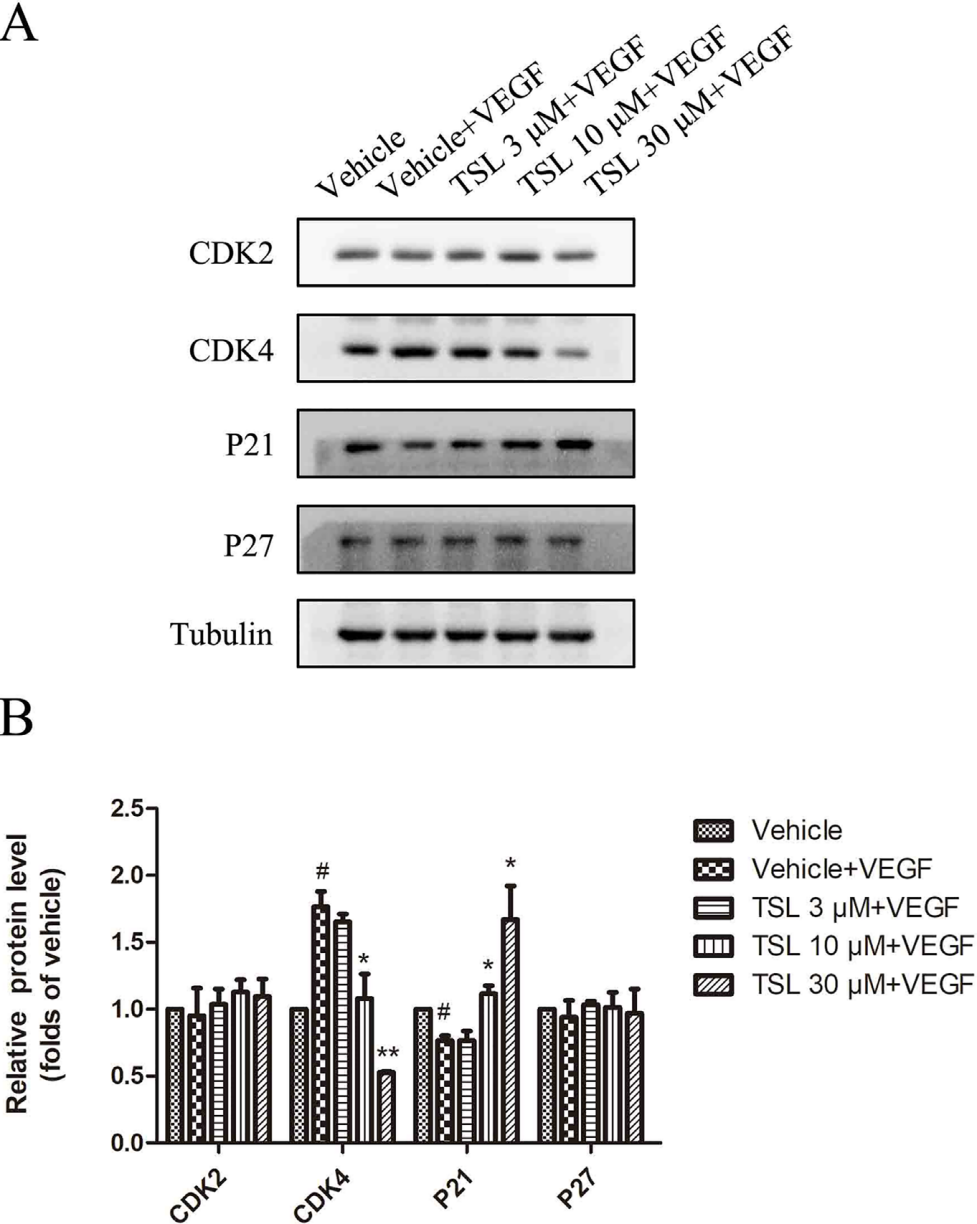
The effect of TSL on cell cycle protein. **(A)** HUVECs were seeded in six-well plates and grown to 80% confluence. Then cells were cultured in M199 and 2% FBS and pretreated with indicated concentrations TSL for 30 min, followed by the addition of VEGF (25 ng/ml) for another 24 h, CDK2, CDK4, P21, P27 was determined by Western blots. **(B)** Quantification of the Western blots. Data are represented as mean ± SEM (n = 3). ^#^ *P* < 0.05 versus the vehicle control. *P < 0.05, **P < 0.01 versus the vehicle + VEGF group.

Moreover, we detected the expression of cell cycle protein CDK2, CDK4, P21, and P27. The results showed that CDK4 was increased and P21 was decreased by VEGF stimulation, whereas the TSL-treated groups partly blocked the effects. However, the CDK2 and P27 levels did not show a significant change (**Figure 3**).

To clarify the effect of TSL on cell cycle progression in HUVECs, we performed flow cytometric assay to examine the DNA content of nuclei. As shown in **Supplement Figure 2**, cells stimulated with VEGF markedly induced G1-S phase transformation. The number of cells in G0G1 was the lowest in the VEGF-stimulated group, and the number of G0G1 phase cells increased from 69.9% to 73.53%, 83.15%, and 82.52% in the presence of 3, 10, 30 µM TSL, and 5 µM cabozanitib, respectively. However, the number of S phase cells decreased from 25.06% to 20.12%, 9.47%, and 14.49%. Taken together, these results suggest that TSL inhibits cell proliferation and prevents cell cycle progression at G1 phase.

### TSL Inhibited the Migration and Invasion of HUVECs Induced by VEGF

**Figure 4:**
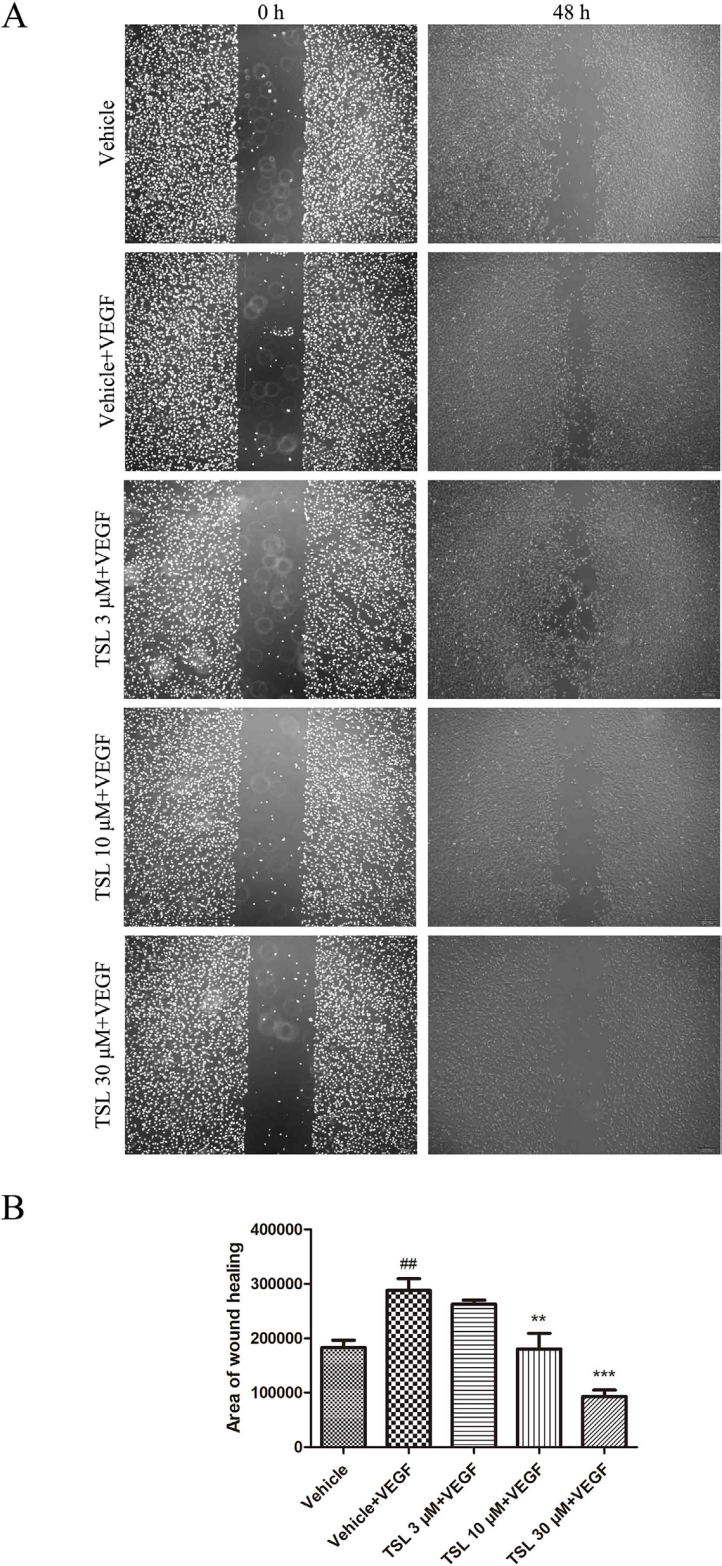
TSL inhibits VEGF induced HUVEC migration. **(A)** In starvation condition, cell monolayers were scratched and treated with vehicle or indicated concentrations of TSL in the presence of VEGF (25 ng/ml) for 48 h. The area of migration into the scratch was then determined. Cells were photographed under phase contrast after 48 h. **(B)** Quantification of the area of wound healing. Data are represented as mean ± SEM (n = 3). ^##^ *P* < 0.01 versus the vehicle group; **P < 0.01, ***P < 0.001 versus the vehicle + VEGF group.

We then assessed the function of TSL in migration of HUVECs by wound healing assay *in vitro*. Exposed to the stimulation of VEGF, a certain amount of cells migrated into the wound area. Similarly, when pretreated with TSL, the migration induced by VEGF was suppressed, 3 μM of TSL had no effect but 10 and 30 μM of TSL suppressed the migration obviously (**Figure 4**). What is more, HUVECs pretreated with 5-µM cabozantinib decreased the amount of cells that migrated into the wound area, the same as the 30-µM TSL group (**Supplement Figure 3**).

**Figure 5:**
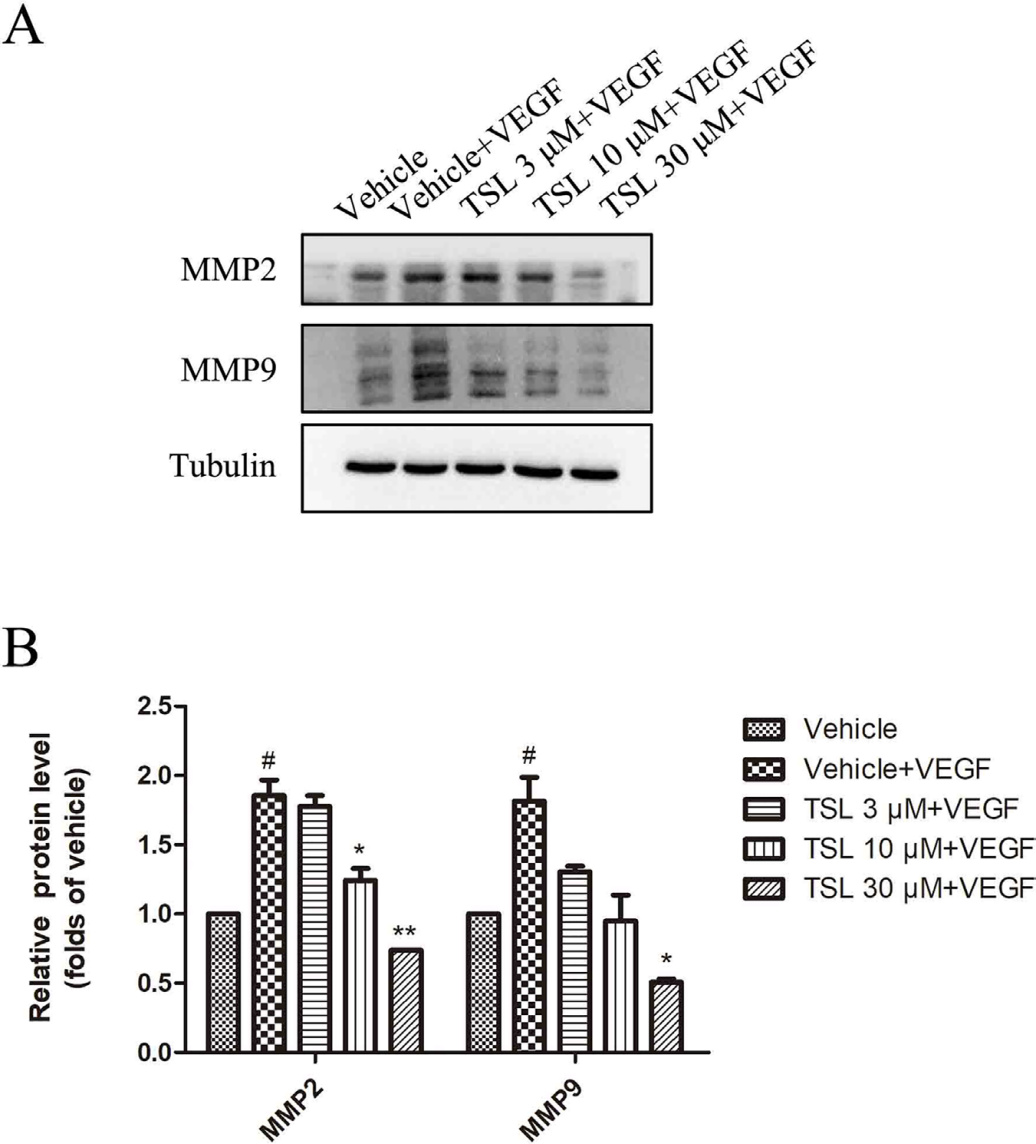
The effects of TSL on matrix metalloproteinases. **(A)** HUVECs were seeded in six-well plates and grown to 80% confluence. Then, the cells were cultured in M199 and 2% FBS and pretreated with indicated concentrations TSL for 30 min, followed by the addition of VEGF (25 ng/ml) for another 24 h, MMP2, MMP9 was determined by Western blots. **(B)** Quantification of the Western blots. Data are represented as mean ± SEM (n = 3). ^#^ *P* < 0.05 versus the vehicle control. *P < 0.05, **P < 0.01 versus the vehicle + VEGF group.

Moreover, we detected migration-associated protein MMP2 and MMP9, which were increased by VEGF stimulation, whereas the TSL-treated groups partly blocked the effects (**Figure 5**). We also detected the effect of TSL on invasion of HUVEC, and the results showed that TSL significantly inhibited the invasion of HUVEC (**Supplement Figure 4**).

### TSL Decreased the Tube Formation of HUVECs Induced by VEGF

**Figure 6:**
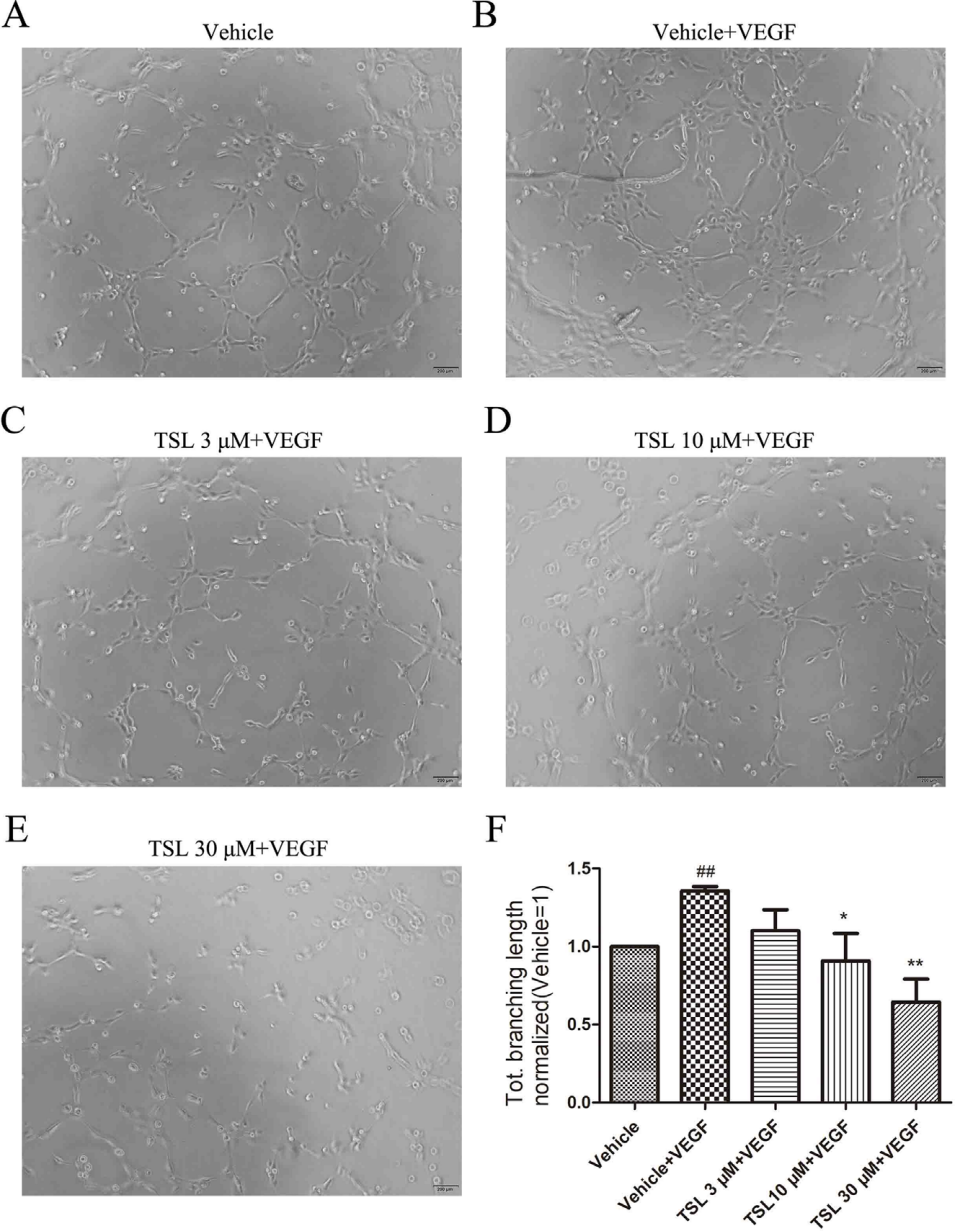
TSL suppresses HUVEC tube formation. **(A-E)** HUVECs were pretreated with indicated concentrations TSL for 6 h, then cells were seeded on matrigel in the presence of VEGF (25 ng/ml) with or without TSL. Cells were photographed after 4 h. **(F)** Quantification of the total branching length (normalized to vehicle). Data are represented as mean ± SEM (n = 3). ^##^ *P* < 0.01 versus the vehicle group; *P < 0.05, **P < 0.01 versus the vehicle + VEGF group.

Tube formation assay was used to evaluate the ability of HUVECs to form capillary-like structures. Compared with the vehicle group, the cells treated with VEGF formed more complex and branched capillary-like structures, whereas the cells treated with TSL blocked the effects partly. The 3-μM TSL-treated group showed very little effect; however, the 10- and 30-μM TSL-treated groups significantly decreased the tube formation induced by VEGF (**Figure 6**).

### TSL Inhibited the VEGFR-2 Signal Pathway Activation and ROS Production of HUVECs Induced by VEGF

**Figure 7:**
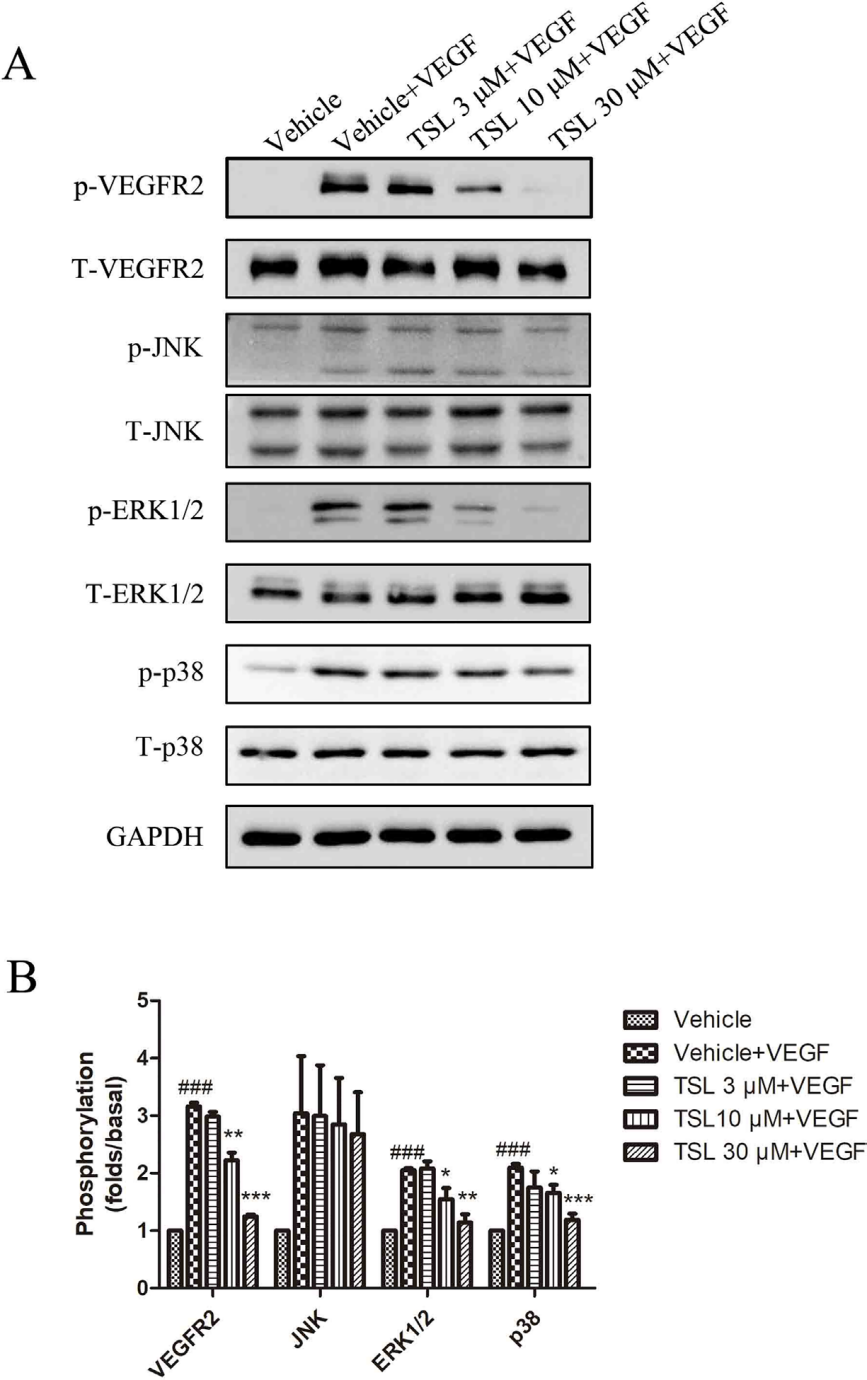
TSL suppresses the VEGFR2 signaling pathway. **(A)** HUVECs were pretreated with indicated concentrations of TSL for 30 min, followed by the addition of VEGF (25 ng/ml) for another 5 min. Phosphorylation status of VEGFR2, JNK, ERK1/2, p38 was determined by western blots. **(B)** Quantification of the Western blots. Data are represented as mean ± SEM (n = 3). ^###^ *P* < 0.001 versus the vehicle control. *P < 0.05, **P < 0.01, ***P < 0.001 versus the vehicle + VEGF group.

VEGFR2 is known as the key signaling receptor of VEGF and the key role in mediating angiogenesis. We measured the protein expressions of VEGFR2, including the total and phosphorylation expressions. The results showed that the stimulation of VEGF increased the expression of phosphorylated VEGFR2, whereas the total VEGFR2 expression had no change. When pretreated with TSL, the expression of phosphorylated VEGFR2 was decreased, which was most obviously at the concentration of 30 μM. Moreover, we detected the total and phosphorylation expressions of ERK1/2, p38, and JNK, respectively. For these three proteins, with no changes in the expressions of total protein, both the phosphorylated protein levels of ERK1/2 and p38 were increased by VEGF stimulation, whereas the phosphorylated JNK level did not show a significant change. Also, the TSL-treated groups blocked the effects partly (**Figure 7**). ROS is known to be a downstream signaling after VEGFR2 activation; therefore, we detected the ROS levels. The results showed that TSL significantly inhibited intracellular VEGF-induced ROS production (**Supplement Figure 5**).

### TSL Inhibits Hypoxia-Induced VEGF, PDGF, EGF, HIF-1α Expression

To further identify the effects of TSL on the expression of proangiogenic factors, we detected VEGF, Platelet derived growth factor (PDGF), Epidermal Growth Factor (EGF), Basic Fibroblast Growth Factor (bFGF), and Hypoxia-Inducible Factor-1α (HIF-1α) in a hypoxia condition to imitate tumor microenvironment. The results showed that TSL inhibits hypoxia-induced VEGF, PDGF, EGF, HIF-1α expression, whereas bFGF level did not show a significant change (**Supplement Figure 6**).

### TSL has no Effect on PI3K/AKT/mTOR and NF-κB Signaling Pathway in HUVECs Induced by VEGF

To explore more mechanism between TSL and angiogenesis, we further measured the protein expression of total and phosphorylated AKT and mammalian target of rapamycin (mTOR). The results showed that the stimulation of VEGF increased the expression of phosphorylated AKT and phosphorylated mTOR, whereas the total AKT and total mTOR expression had no obvious change. When pretreated with 30-µM TSL and 5-µM cabozanitib, the expression of phosphorylated and total AKT and mTOR had no obvious change compared with the VEGF-stimulated group (**Supplement Figure 7**). TSL has also been known to have anti-inflammatory effect, and VEGF signaling is also involved in inflammation. To further clarify the mode of action of TSL, we checked the role of TSL in VEGF-stimulated p-p65 expression. The results showed that the level of p-p65 did not show any obvious change (**Supplement Figure 8**).

### TSL Inhibits Angiogenesis *In Vivo*

**Figure 8:**
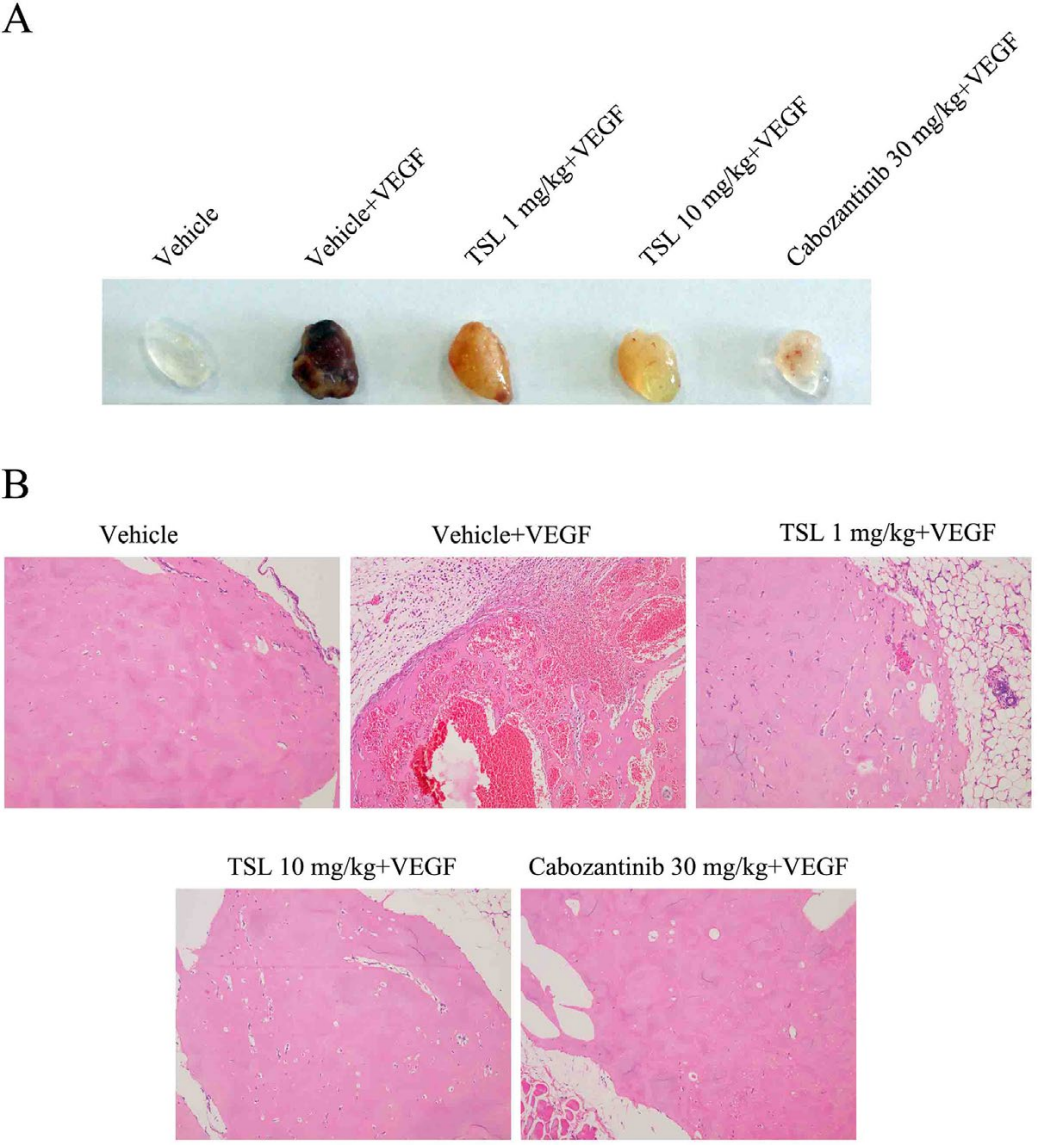
Inhibitory effect of TSL on VEGF induced *in vivo* neovascularization. Matrigel were mixed with 50 units/mL heparin in presence or absence of VEGF (100 ng/mL). The mixed matrigel were subcutaneously injected into the flank abdomen of mice. After 7 days, the matrigel plugs were separated from euthanized mice. **(A)** Representative photos and **(B)** histological images are shown.

To evaluate the effect of TSL on angiogenesis *in vivo*, Matrigel plug assay, which specifically and directly reflects the neo-vascularization potential, was performed. Matrigel alone or Matrigel mixed with VEGF was subcutaneously injected to mice, and Matrigel plugs were harvested 1 week later. The photo of Matrigel plugs showed that VEGF-stimulated vascularization was markedly suppressed by TSL (1 and 10 mg/kg) and cabozanitib (30 mg/kg) treatment (**Figure 8A**). Consistently, the H&E staining images from Matrigel plugs further proved that TSL (1 and 10 mg/kg) and cabozanitib (30 mg/kg) treatment significantly reduced VEGF-stimulated density of microvessels and the invading of ECs (**Figure 8B**).

## Discussion

Angiogenesis plays an important role in many diseases described as before, especially in the tumor growth and maintain the metastasis, even observed in the early period of tumor progression. Followed by deterioration of basement membrane, endothelial cells move into the stromal space, proliferate, and assemble into tubes, matrix also participate this process (Moon et al., 2010; Wong and Crawford, 2013; Eelen et al., 2015). The related regulator of angiogenesis include VEGF, Delta-like 4 (Dll4)/Notch, Wnt, and dimethylarginine dimethylaminohydrolase/asymmetric dimethylarginine (DDAH/ADMA) pathways, and the VEGF family is the most important one among of them (Hong et al., 2018). The VEGF family includes VEGFA, VEGFB, VEGFC, VEGFD, and placental growth factor (PlGF). Studies are focus on VEGFA which used as an indicator for poor prognosis, bind to both VEGF receptor 1 (VEGFR-1) (Flt-1) and -2 (VEGFR-2) (KDR/Flk-1), lead to activation of phospholipase C (PLC), PKC, and MAP-kinase (Kim et al., 2010; Das et al., 2016). Therefore, VEGF signal pathway inhibitors have been identified as the new targets by inhibition of tumor angiogenesis with low drug toxicity and little adverse events, such as bevacizumab and ranibizumab.

TSL was used as a traditional Chinese medicine extracted from the flower buds of *T. farfara* (Kuandonghua) to cure respiratory diseases, and presented anti-inflammatory and antioxidant effects in previous studies. There were clues that TSL suppresses colon cancer cell proliferation by inhibition of β-catenin-dependent Wnt pathway and decreased the expression of cyclin D1 and c-myc (Li et al., 2014), but there was no research about TSL in angiogenesis so far. In the present study, we observed that TSL at concentrations of 10 and 30 µM inhibited HUVEC cell proliferation, cell migration, and tube formation induced by VEGF, which are necessary factors in angiogenesis. Also, TSL inhibited VEGF-induced angiogenesis revealed by Matrigel plug assay *in vivo*. These results revealed that TSL possessed the anti-angiogenic activity. Further, our results demonstrated that the anti-angiogenic activity of TSL was based on the inhibition of VEGF-VEGFR2 signal pathway. VEGFR2 is the main signal transducing VEGF receptor for angiogenesis and mitogenesis of endothelial cells. After receptor dimerization and autophosphorylation, several signal transduction molecules are activated, such as ERK and p38 (Cross et al., 2003). ERK pathway plays a crucial role in VEGFA mitogenic signaling and promote angiogenesis by acting as a major participant in the regulation of cell growth and proliferation (Cross et al., 2003). p38 pathway conveys the VEGF signal to microfilaments, inducing rearrangements of the actin cytoskeleton that regulate endothelial cell migration and promote angiogenesis. Our results revealed that TSL dose dependently inhibited the expression of p-VEGFR2 and also reduced the expression of p-ERK1/2 and p-p38, but not impact the JNK activity. Moreover, we also detected PI3K/AKT/mTOR signaling pathway, and the result showed that TSL had no effect on PI3K/AKT/mTOR signaling pathway in HUVECs induced by VEGF.

**Figure 9:**
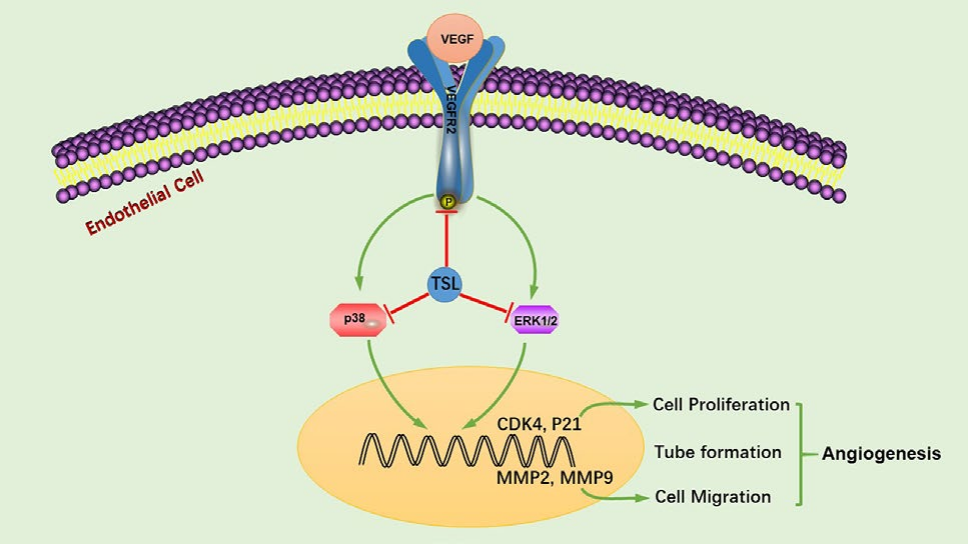
TSL suppresses angiogenesis by inhibiting the VEGFR2 signaling pathway. TSL inhibit VEGF-induced VEGFR2 phosphorylation, sequentially inhibiting the levels of p-ERK and p-p38, additionally increasing the gene expression of P21 and decreasing the gene expression of CDK4, MMP2, and MMP9, consequently, inhibiting endothelial cell proliferation, migration, tube formation, and angiogenesis.

In conclusion, we showed that TSL inhibit VEGF-induced VEGFR2 phosphorylation, sequentially inhibiting the levels of p-ERK and p-p38, also increasing the gene expression of P21, and decreasing the gene expression of CDK4, MMP2, and MMP9, consequently inhibiting endothelial cell proliferation, migration, tube formation, and angiogenesis (**Figure 9**). Based on these findings, we suggest that TSL may serve as potential scaffolds for the development of therapeutic agents to treat angiogenesis-dependent diseases. 

## Author Contributions

ML and YaW conceived and designed the experiments. JL, JP, ML, and YaW performed the experiments and prepared the article. SZ, YZ, YiW, JH, CZ, MC, GX, YH, and KH participated in discussions of data analysis. CZ performed the H&E staining images and analysis. ML and YaW prepared and revised the article. All authors gave final approval.

## Funding

This study has been supported by the National Natural Science Foundation of China (81300100), Research fund of union hospital (02.03.2017-23), Hubei key laboratory of biological targeted therapy research open fund (02.03.2018-62), and Integrated Innovative Team for Major Human Diseases Program of Tongji Medical College, HUST.

## Conflict of Interest Statement

The authors declare that the research was conducted in the absence of any commercial or financial relationships that could be construed as a potential conflict of interest.
