# Supplementary material for: Tussilagone Suppresses Angiogenesis by Inhibiting the VEGFR2 Signaling Pathway
Source: Front Pharmacol. 2019 Jul 5;10:764. doi: 10.3389/fphar.2019.00764 (PMC6624780; doi:10.3389/fphar.2019.00764)

Materials and Methods

EdU incorporation assay

Cell proliferation of HUVECs was determined by EdU incorporation assay. HUVECs were seeded in 96-well plates (1.2×10^4 cells/well). Cells were pretreated with TSL 30 μM and Cabozantinib (XL184) 5 μM for 30 min in ECM containing 10% FBS, then VEGF (25 ng/mL) was added into each group for another 12 h. Next, DNA synthesis was detected by EdU kit.

Would healing assay

The HUVECs were cultured into 6-well plates up to 80% confluence. Cell monolayers were wounded with a sterile 10 μL pipette tip and washed with PBS for twice. Cells were then cultured in ECM containing 10% FBS and treated with TSL at 30 μM and Cabozantinib (XL184) 5 μM in the absence or presence of VEGF (25 ng/mL) for 24 h. Cells were photographed using Olympus cellSens Entry, the rate of cell migration was determined by the area of migration into the scratch using an Image J program.

Cell cycle assay

Cell cycle was determined using propidium iodide (PI) staining by flow cytometry. Cells were cultured in ECM containing 10% FBS and treated with TSL at various concentrations and Cabozantinib (XL184) 5 μM in the absence or presence of VEGF (25 ng/mL) for 24 h. Cells were harvested and washed twice in cold PBS, then fixed in 70 % cold alcohol overnight at 4 °C, then washed twice in cold PBS, cells were calculated with the same numbers about 1×10^6, then stained with PI solution at 4 °C in the dark for 30 min. The cells were analyzed by flow cytometry (BD FACSCalibur, BD Bioscience, San Diego, CA, USA), data were analyzed using ModFit Software. The experiment was repeated three times.

Transwell assay

A 24-well transwell plate (Corning, UK) containing 8.0 μm polycarbonate membrane

was coated with 0.1 ml 300 ug/ml Matrigel (BD Biosciences) in 37°C for 2 h, cells were pretreated with TSL or cabozantinib with or without VEGF for 12 h. Then, 2× 10^4 cells were seeded onto the upper chambers. After 12 h of incubation, the cells that failed to pass through the membrane were removed, while those succeeded in invading were washed twice with PBS and then fixed with 4% paraformaldehyde and then stained with crystal violet for 15 min. The invading cells were photographed using Olympus cellSens Entry.

Reactive oxygen species (ROS) measurement

Measurement of reactive oxygen species 2'7'-Dichlorofluorescein diacetate (DCFH-DA, Beyotime) was used to measure ROS formation. HUVECs were incubated in 10 μM DCFH-DA at 37°C for 40 min, then cells were washed with PBS three times to remove DCFH-DA that not entered in cells. After that, HUVECs were exposed to different concentrations of TSL with or without VEGF for 1 h. The fluorescence was visualized immediately at wave lengths of 488 nm for excitation and 525 nm for emission by inverted fluorescence

Western blotting

The HUVECs were seeded into 6-well plates up to confluence. After pretreated with TSL 30 μM and Cabozantinib (XL184) 5 μM for 30 min, the cells were stimulated by VEGF (25 ng/mL) for another 10 min or were incubated in hypoxia condition (37°C, 1% O_2) respectively for 12h. Western blotting was performed according to the procedures described previously.

Figure legend

Supplement Figure 1

TSL inhibits VEGF induced HUVEC proliferation. (A) HUVECs were pretreated with 30 μM TSL and 5 μM cabozanitib for 30 min, then VEGF (25 ng/mL) was added into each group for 12 h. The cell were stained with EdU (red) and DAPI (blue). Representative images were showed. (B) Quantification of the proliferation cell

(normalized to vehicle). Data are represented as mean ± SEM (n=3). $^{\#}$P < 0.05 versus the vehicle group; *P < 0.05, **P < 0.01 versus the vehicle + VEGF group.

Supplement Figure 2

The effects of TSL on cell cycle. (A) HUVECs were pretreated with indicated concentration of TSL and cabozanitib with or without the presence of VEGF (25 ng/mL) for 24 h. Subsequently, cell cycle status was analyzed by flow cytometry. The number of cells in each phase were displayed on the picture. (B) Quantification of the cells in S phase. Data are represented as mean ± SEM (n=3). $^{\#\#\#}$P < 0.001 versus the vehicle group; *P < 0.05 versus the vehicle + VEGF group.

Supplement Figure 3

TSL inhibits VEGF induced HUVEC migration. (A) Cell monolayers were scratched and treated with vehicle or 30 μM TSL and cabozanitib in the presence of VEGF (25 ng/mL) for 24 h. Cells were photographed under phase contrast after 24 h. (B) Quantification of the area of wound healing. Data are represented as mean ± SEM (n=3). $^{\#\#}$P < 0.01 versus the vehicle group; **P < 0.01, ***P < 0.001 versus the vehicle + VEGF group.

Supplement Figure 4

TSL inhibits VEGF induced HUVEC invasion. (A) HUVECs were pretreated with 30 μM TSL and cabozantinib for 30 min, then VEGF were added to HUVEC for 12 h. After fixation, the invading cells were stained with crystal violet for 15 min and were photographed. (B) Quantification of the invasion cell number. Data are represented as mean ± SEM (n=3). #P < 0.05 versus the vehicle group; **P < 0.01 versus the vehicle + VEGF group.

Supplement Figure 5

TSL inhibits VEGF induced ROS production. (A) HUVECs were incubated in 10 μM DCFH-DA at 37°C for 40 min, then exposed to different concentrations of TSL with

or without VEGF for 1 h. The fluorescence was visualized immediately by inverted fluorescence microscope. (B) Quantification of the fluorescence intensity. Data are represented as mean ± SEM (n=3). $^{###}P < 0.001$ versus the vehicle group; $*P < 0.05$, $**P < 0.01$ versus the vehicle + VEGF group.

Supplement Figure 6

The effect of TSL on VEGF, PDGF, EGF, HIF-1α expression in hypoxia condition. (A) The HUVECs were pretreated with TSL or cabozantinib 30 min. Then incubated in a standard incubator (37°C, 5% CO2) or in hypoxia condition (37°C, 1% O_2) respectively for 12 h. Western blots were performed. (B) Quantification of the western blots. Data are represented as mean ± SEM (n=3). $^{\#}P < 0.05$, $^{\#\#}P < 0.01$, $^{\#\#\#}P < 0.001$ versus the vehicle control. $*P < 0.05$, $**P < 0.01$ versus the vehicle + hypoxia control.

Supplement Figure 7

TSL have no obvious effect on PI3K/AKT/mTOR signaling pathway. (A) HUVECs were pretreated with 30 μM TSL and 5 μM cabozanitib for 30 min, followed by the addition of VEGF (25 ng/mL) for another 10 min. Phosphorylation status of AKT and mTOR was determined by western blots. (B) Quantification of the western blots. Data are represented as mean ± SEM (n=3). #P < 0.05 versus the vehicle group.

Supplement Figure 8

The effect of TSL on NF-κB signaling pathway. (A) The HUVECs were pretreated with 30 μM TSL and cabozantinib, then stimulated by VEGF for 30 min to detect the expression of p-p65 protein. (B) Quantification of the western blots. Data are represented as mean ± SEM (n=3).

Supplement Figure 1

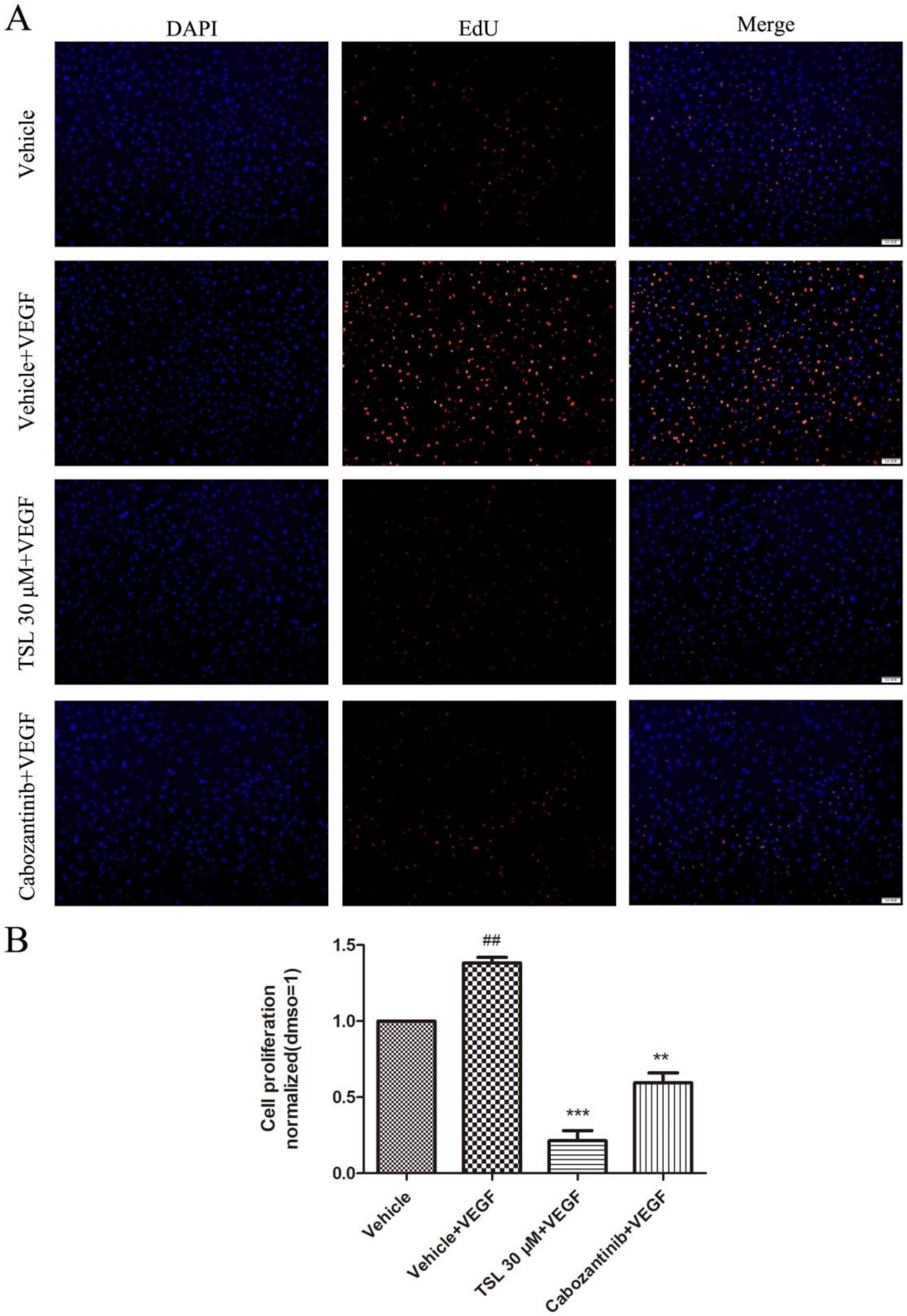

Supplement Figure 2

A

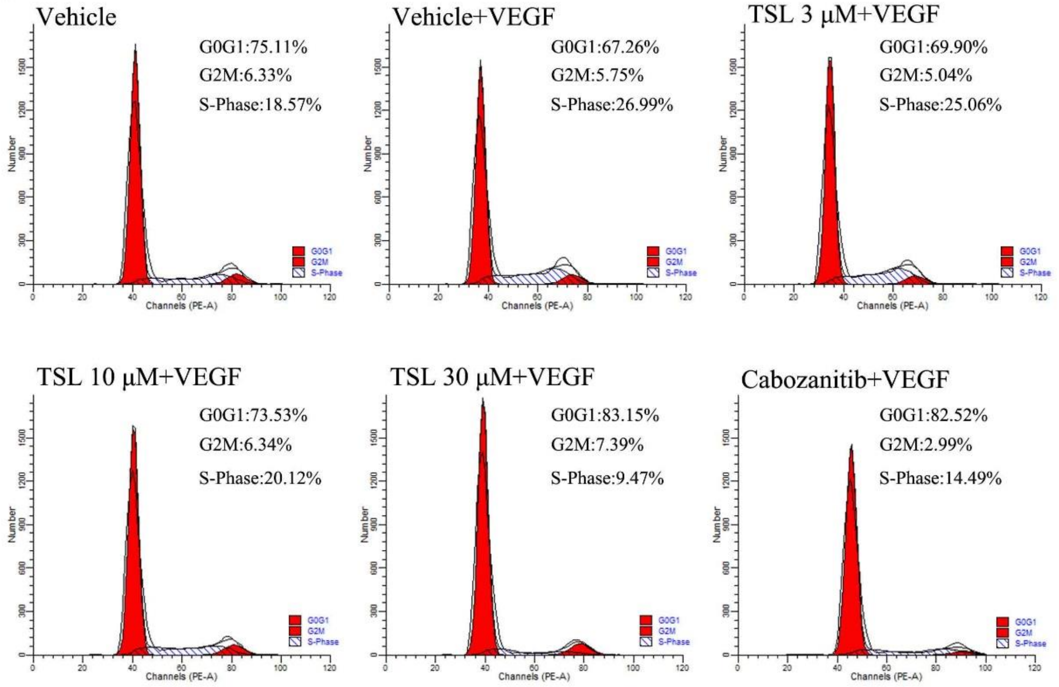

B

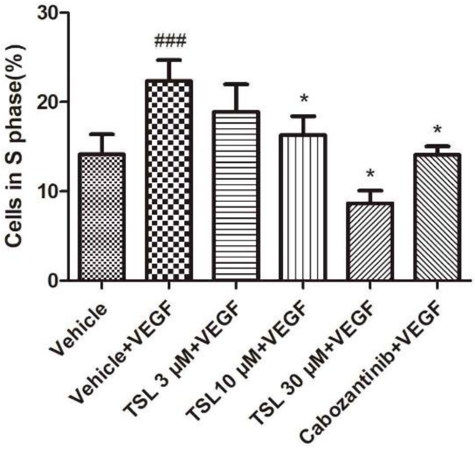

Supplement Figure 3

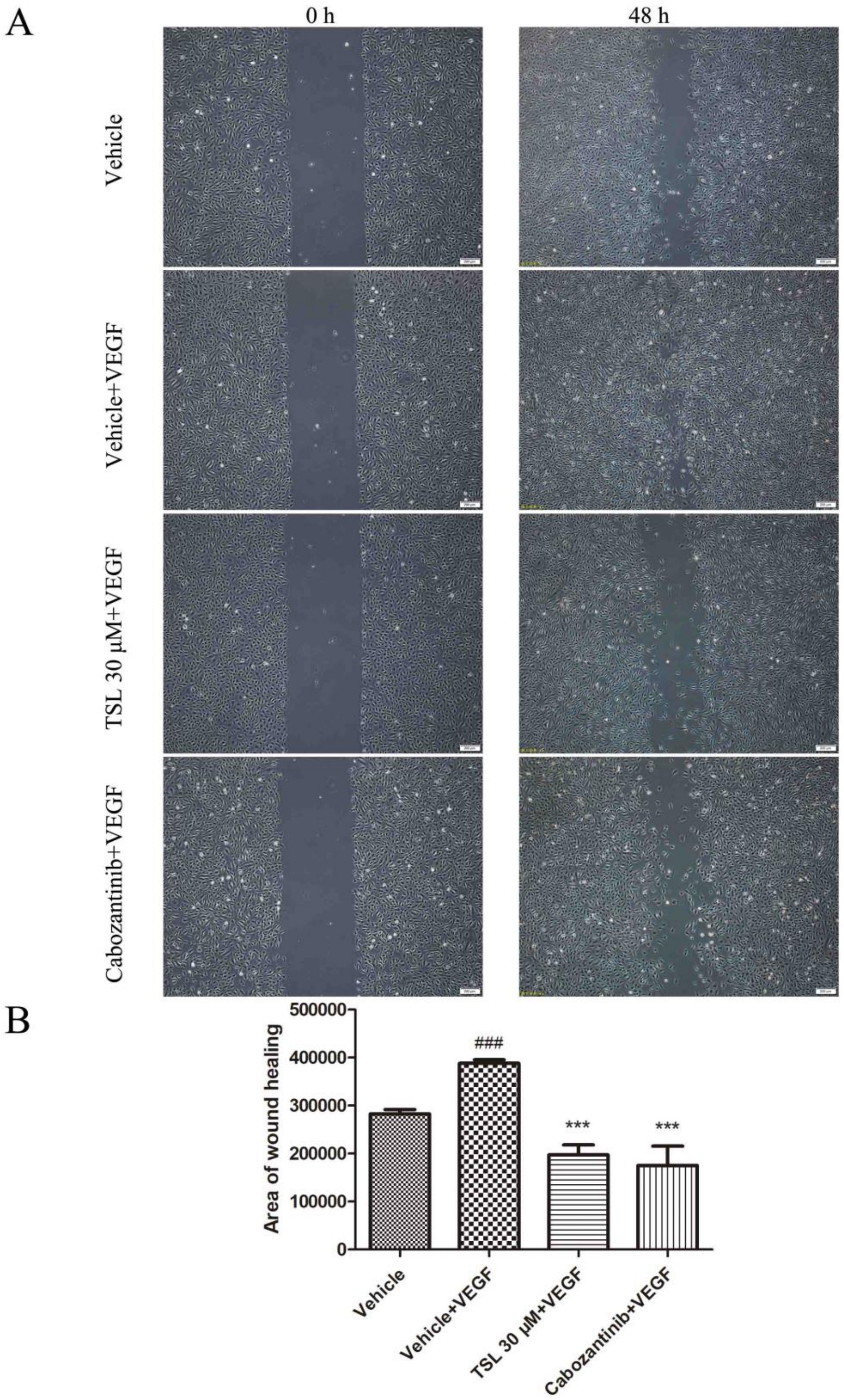

Supplement Figure 4

A

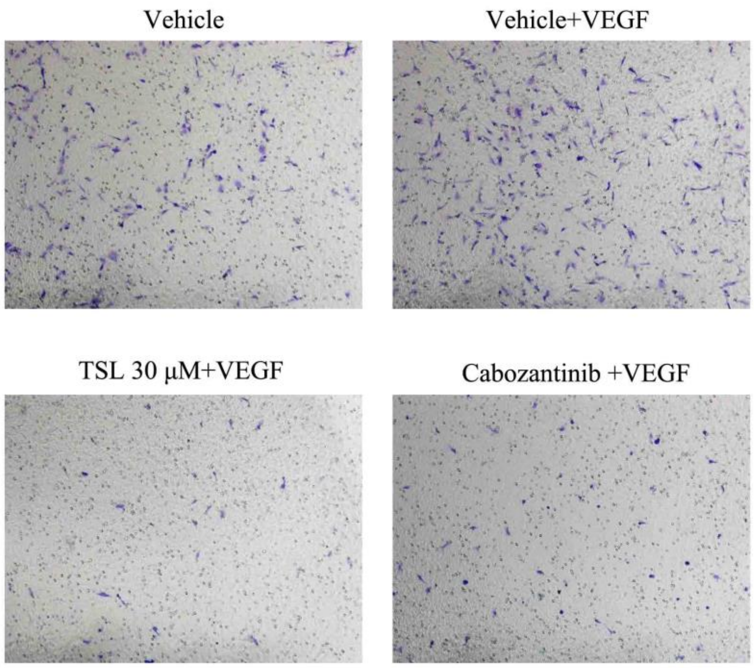

B

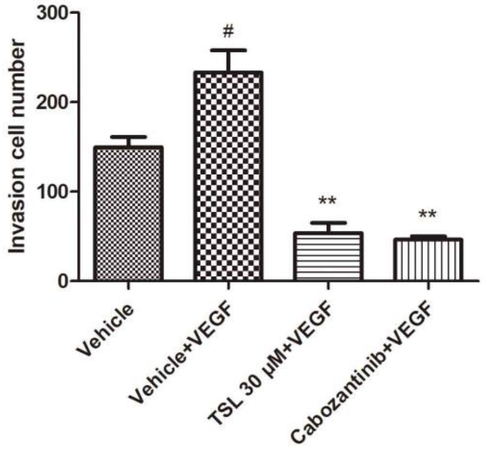

Supplement Figure 5

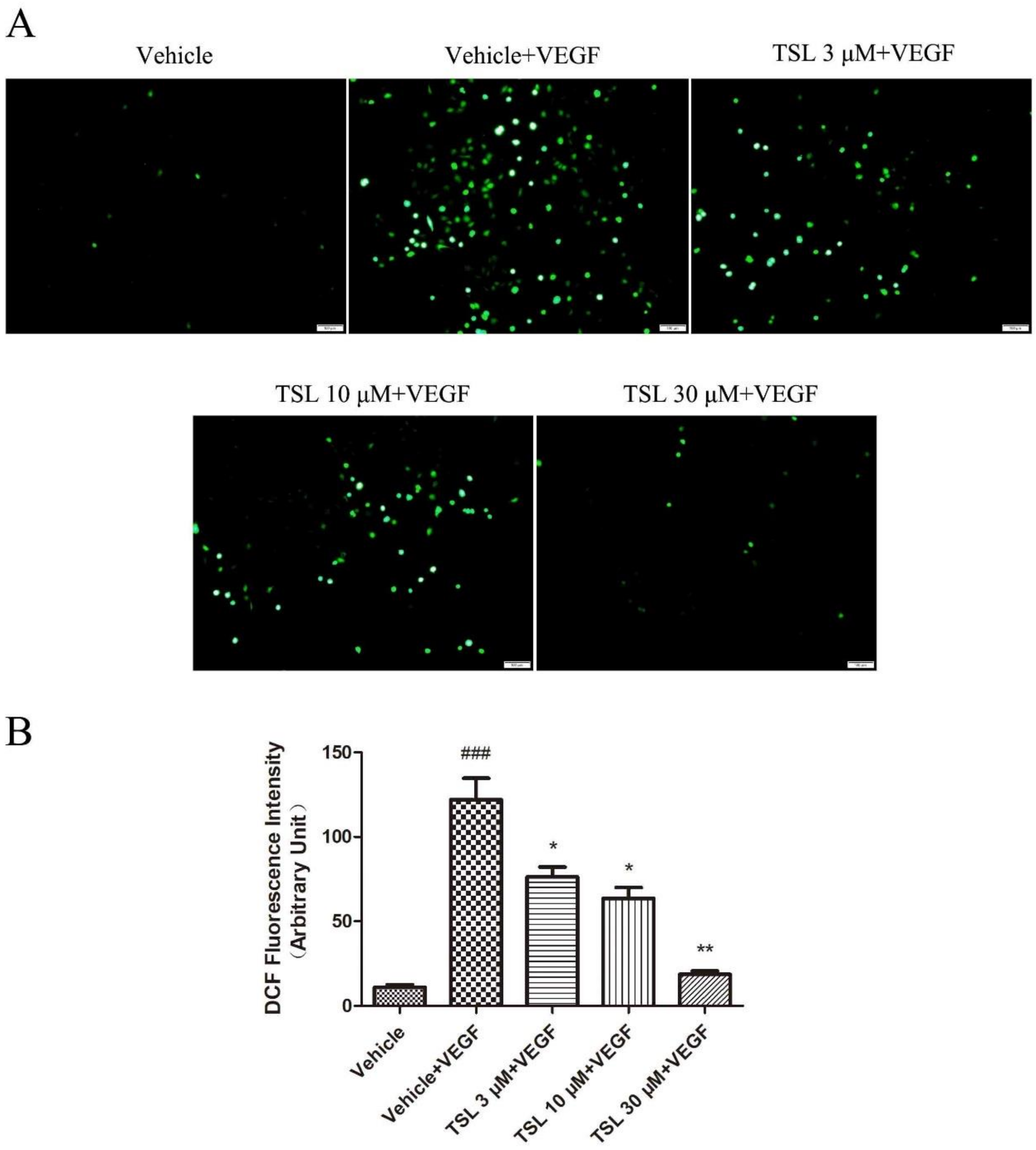

Supplement Figure 6

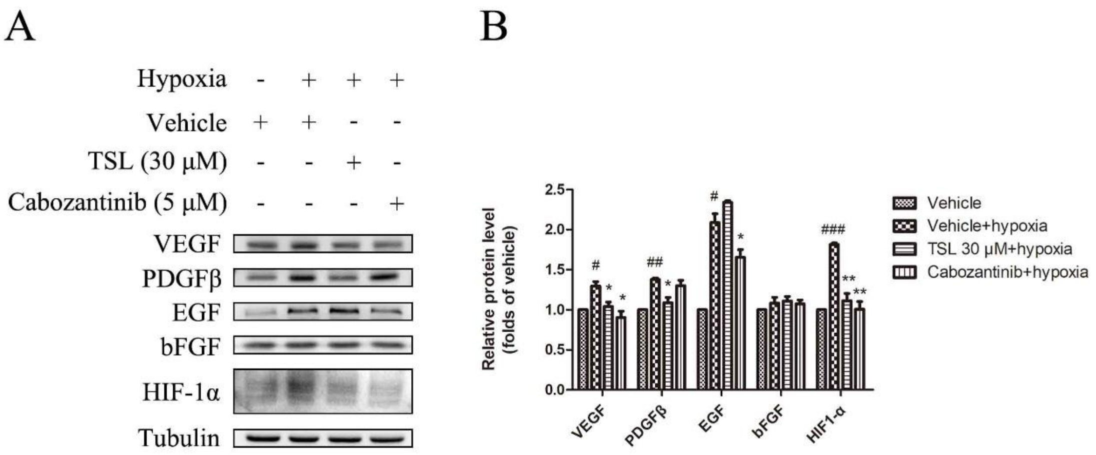

Supplement Figure 7

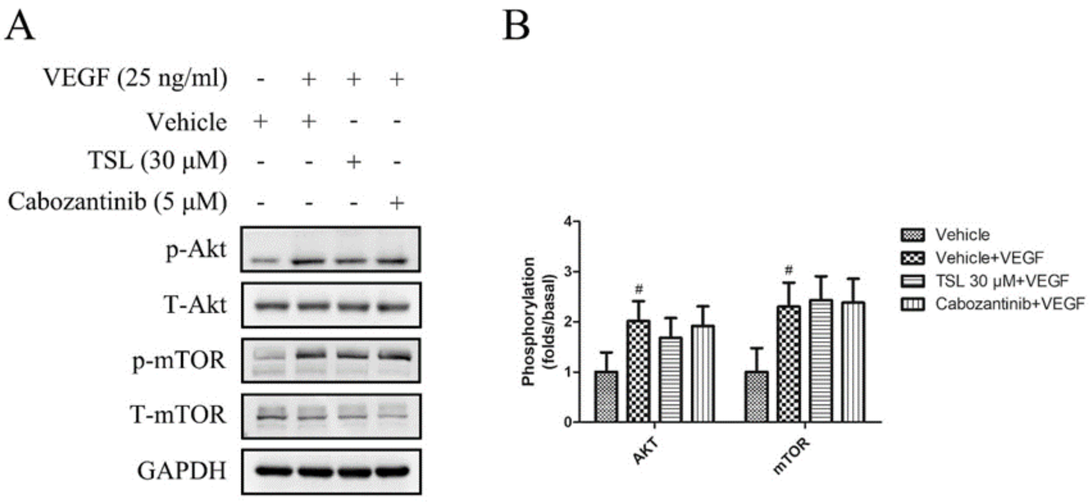

Supplement Figure 8

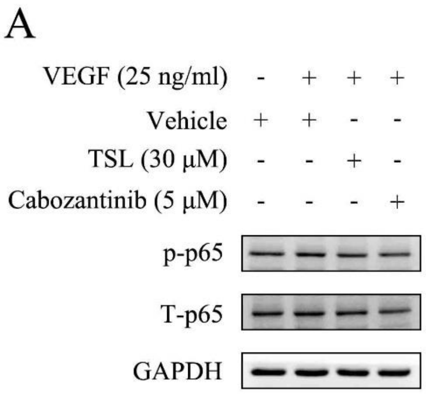

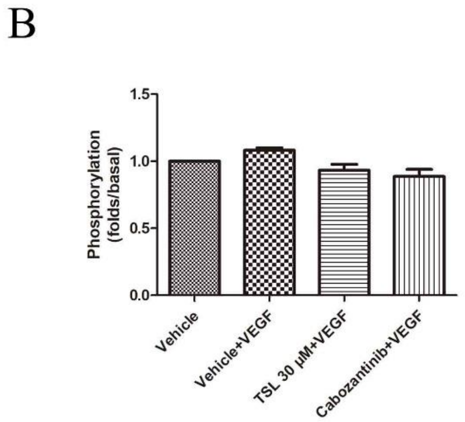

Supplement: Supplementary file 1 [file DataSheet_1.pdf]
